# Supplementary material for: What about Current Diversity of Mycolactone-Producing Mycobacteria? Implication for the Diagnosis and Treatment of Buruli Ulcer
Source: Int J Mol Sci. 2023 Sep 6;24(18):13727. doi: 10.3390/ijms241813727 (PMC10531242; doi:10.3390/ijms241813727)

Supplementary Figures

Supplementary Figure S1. Presence and copy number of each MIRU-VNTR loci identified in the literature and previously used to discriminate *M. ulcerans* strains from different geographic origins. MPM variants were selected based on the availability of complete genomes. *M. marinum* strains were selected as control groups since they do not belong to the MPM complex. *M. gilvum* was also included as an outgroup since this mycobacterium does not belong to the MPM complex although it was found in BU cases in French Guiana (Combe et al. 2020). The color gradient indicates the presence/absence of each MIRU-VNTR in the genome of each selected mycobacterium and numbers indicate the copy number of each genetic marker. Only the loci amplified by all MPM variants selected here are presented in this figure: MIRU1 has been removed from the analysis because this locus is not amplified in the Surinam and French Guiana *M. ulcerans* strains. We show that MIRU33 (Stragier et al. 2005) cannot discriminate between strains of *M. ulcerans*, *M. shinshuense* and *M. marinum*. MIRU5 (Stragier et al. 2005) cannot discriminate between strains of *M. ulcerans* and *M. shinshuense*. MIRU7 and MIRU9 (Stragier et al. 2005) show the same profile for all variants/species tested. ST1 (Hilty et al. 2006) shows the same number of repeats in almost all MPM variants, except the French Guiana variant and most *M. marinum* strains. VNTR14 and VNTR15 (Ablordey et al. 2005) can only distinguish *M. ulcerans* strains not originating from French Guiana but all MPM variants show the same profile as *M. marinum*. VNTR18 can only identify *M. shinshuense* from other MPM variants whereas VNTR19 has the greatest discriminatory power between MPM variants as it can differentiate *M. ulcerans* from *M. pseudoshottsii* and *M. shinshuense* (Ablordey et al. 2005). VNTR6 is not informative at all (Ablordey et al. 2005). *M. gilvum* does not harbor these MIRU-VNTR loci in its genome, with the exception of MIRU7 (1 copy) and VNTR14 (2 copies) although these markers cannot differentiate it from other MPM variants or from *M. marinum* strains.

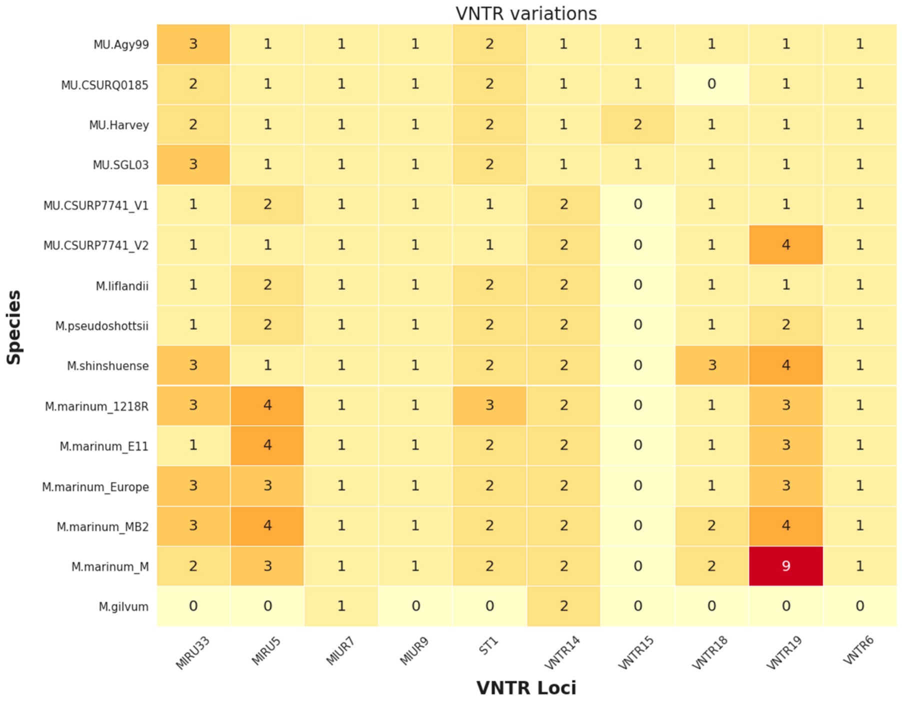

Supplementary Figure S2. Presence and copy number of the 34 VNTR loci identified by Hilty et al. (2007). MPM variants were selected based on the availability of complete genomes. *M. marinum* strains were selected as control groups since they do not belong to the MPM complex. *M. gilvum* was also included as an outgroup since this mycobacterium does not belong to the MPM complex although it was found in BU cases in French Guiana (Combe et al. 2020). The color gradient indicates the presence/absence of each VNTR in the genome of each mycobacteria selected and numbers indicate the copy number of each genetic marker. As shown here many of these loci were not found in the genomes analyzed here. Overall, *M. ulcerans* strains exhibit quite different profiles than other MPM variants and *M. marinum* strains. However, none of the other MPM variants can be distinguished based on these VNTR since they present the same number of repeats than *M. marinum* strains. None of these VNTR were found in the genome of *M. gilvum*.

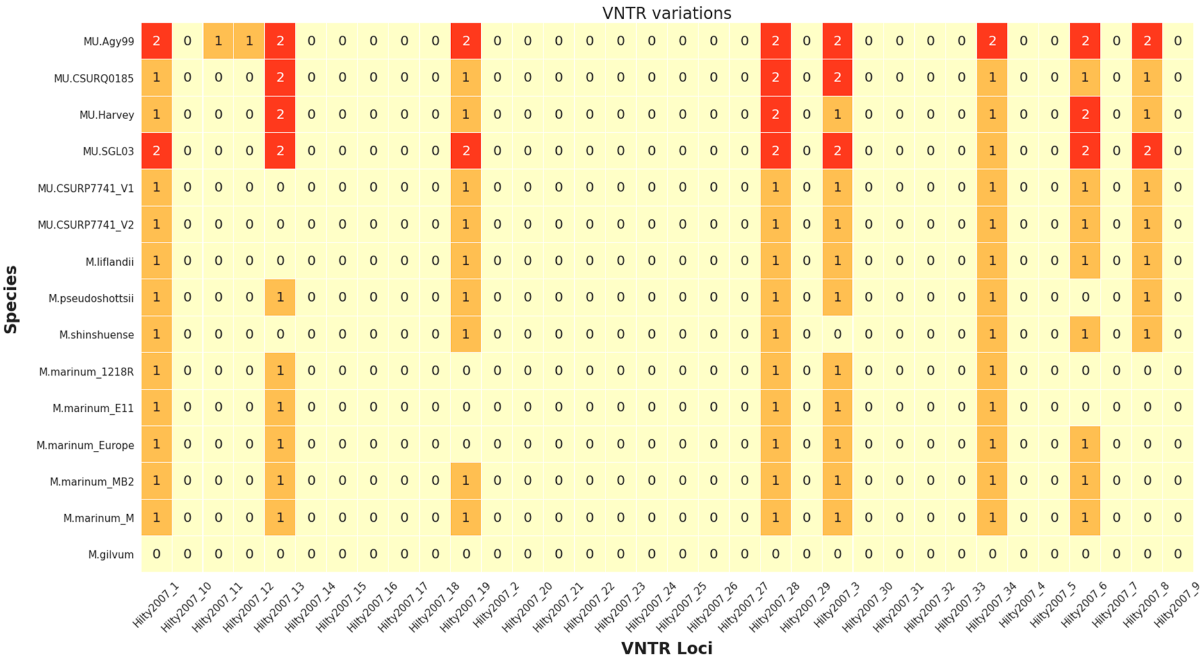

Supplement: Supplementary file 1 [file ijms-24-13727-s001.zip › Supplementary Figures.pdf]
